# *nab*-Paclitaxel-Based Therapy in Underserved Patient Populations: The ABOUND.70+ Study in Elderly Patients With Advanced NSCLC

**DOI:** 10.3389/fonc.2018.00262

**Published:** 2018-07-24

**Authors:** Corey J. Langer, Edward S. Kim, Eric C. Anderson, Robert M. Jotte, Manuel Modiano, Daniel E. Haggstrom, Matei P. Socoteanu, David A. Smith, Christopher Dakhil, Kartik Konduri, Tymara Berry, Teng J. Ong, Alexandra Sanford, Katayoun Amiri, Jonathan W. Goldman, Jared Weiss, on behalf of the ABOUND.70+ Investigators, Ajeet Gajra

**Affiliations:** ^1^Abramson Cancer Center, University of Pennsylvania, Philadelphia, PA, United States; ^2^Levine Cancer Institute, Carolinas Healthcare System, Charlotte, NC, United States; ^3^Knight Cancer Institute, Oregon Health & Science University, Portland, OR, United States; ^4^Rocky Mountain Cancer Centers, Denver, CO, United States; ^5^Arizona Clinical Research Center, Tucson, AZ, United States; ^6^Texas Oncology, Longview, TX, United States; ^7^Compass Oncology, Vancouver, WA, United States; ^8^Cancer Center of Kansas, Wichita, KS, United States; ^9^Baylor Charles A. Sammons Cancer Center, Texas Oncology PA, Dallas, TX, United States; ^10^Celgene Corporation, Summit, NJ, United States; ^11^David Geffen School of Medicine at UCLA, Los Angeles, CA, United States; ^12^Lineberger Comprehensive Cancer Center, University of North Carolina, Chapel Hill, NC, United States

**Keywords:** advanced non-small cell lung cancer, *nab*-paclitaxel, carboplatin, elderly, randomized trial, phase 4

## Abstract

The phase 4 ABOUND.70+ trial assessed the safety and efficacy of *nab*-paclitaxel/carboplatin continuously or with a 1-week break between cycles in elderly patients with advanced non-small cell lung cancer (NSCLC). Patients ≥70 years with locally advanced/metastatic NSCLC were randomized 1:1 to first-line *nab*-paclitaxel days 1, 8, 15 plus carboplatin day 1 of a 21-day cycle (21d) or the same *nab*-paclitaxel/carboplatin regimen with a 1-week break between cycles (21d + break; 28d). The primary endpoint was the percentage of patients with grade ≥ 2 peripheral neuropathy (PN) or grade ≥ 3 myelosuppression. Other key endpoints included progression-free survival (PFS), overall survival (OS), and overall response rate (ORR). A total of 143 patients were randomized (71 to 21d, 72 to 21d + break). The percentage of patients with grade ≥ 2 PN or grade ≥ 3 myelosuppression was similar between the 21d and 21d + break arms (76.5 and 77.1%; *P* = 0.9258). Treatment exposure was lower in the 21d arm compared with the 21d + break arm. Median OS was 15.2 and 16.2 months [hazard ratio (HR) 0.72, 95% CI 0.44–1.19; *P* = 0.1966], median PFS was 3.6 and 7.0 months (HR 0.48, 95% CI 0.30–0.76; *P* < 0.0019), and ORR was 23.9 and 40.3% (risk ratio 1.68, 95% CI 1.02–2.78; *P* = 0.0376) in the 21d and 21d + break arms, respectively. In summary, the 1-week break between treatment cycles significantly improved PFS and ORR but did not significantly reduce the percentage of grade ≥ 2 PN or grade ≥ 3 myelosuppression. Overall, the findings support the results of prior subset analyses on the safety and efficacy of first-line *nab*-paclitaxel/carboplatin in elderly patients with advanced NSCLC.

## Introduction

Treatment of elderly patients with non-small cell lung cancer (NSCLC) can be challenging due to increased comorbidities, decline in organ function, and lower bone marrow reserve, which may lead to altered pharmacokinetics and increased concerns over toxicity compared with younger patients (1). These and other factors may contribute to suboptimal treatment, or, in many cases, to no treatment at all. Although the median age of patients diagnosed with advanced NSCLC is 70 years, elderly patients remain underrepresented in clinical trials (1, 2).

The randomized trials that exist to guide care have progressively intensified the treatment of elderly patients with advanced NSCLC, improving survival but at the cost of toxicity. The phase 3 ELVIS trial demonstrated superior survival and quality of life (QoL) with single-agent vinorelbine compared with best supportive care (3). Platinum doublets have since become a standard of care in elderly patients with advanced NSCLC. The phase 3 IFCT-0501 trial compared paclitaxel and carboplatin combination therapy with single-agent vinorelbine or gemcitabine in patients ≥70 years (4). Of note, paclitaxel was given weekly on days 1, 8, and 15, with a break on day 22, and the full dose of carboplatin was administered on day 1. Progression-free survival (PFS) and overall survival (OS) were significantly improved with combination treatment [median OS 10.3 vs 6.2 months, hazard ratio (HR) 0.64, 95% CI 0.52–0.78, *P* < 0.0001; median PFS 6.0 vs 2.8 months, HR 0.51, 95% CI 0.42–0.62, *P* < 0.0001] (4). The frequency of grade 3 or 4 toxicity was also increased—neutropenia, febrile neutropenia, thrombocytopenia, sensory neuropathy, and anemia were more commonly observed in the paclitaxel/carboplatin arm than the single-agent arms. In a phase 3 study comparing *nab*-paclitaxel/carboplatin with paclitaxel/carboplatin, 15% of the intent-to-treat population was elderly (≥70 years) (5). In this subgroup, survival was significantly increased with *nab*-paclitaxel/carboplatin vs paclitaxel/carboplatin (median OS 19.9 vs 10.4 months, HR 0.583, 95% CI 0.39–0.88; *P* = 0.009). A significantly lower percentage of patients aged ≥70 years treated with *nab*-paclitaxel/carboplatin experienced neutropenia, neuropathy, and arthralgia (all *P* < 0.05), but a higher percentage experienced anemia vs patients treated with paclitaxel/carboplatin (*P* < 0.05). Recently, this benefit has appeared to extend to more vulnerable patients with NSCLC. Zukin et al. demonstrated improved outcomes with pemetrexed and carboplatin vs pemetrexed in a subset of elderly patients with NSCLC and performance status (PS) 2 (6).

The phase 4 ABOUND.70+ study was designed to prospectively evaluate *nab*-paclitaxel/carboplatin in elderly patients with advanced NSCLC and to investigate the merits of introducing a 1-week break at the end of each treatment cycle, thereby replicating the schedule used in the IFCT-0501 trial, which demonstrated a survival advantage for paclitaxel/carboplatin (4).

## Materials and Methods

### Study Population

Patients with histologically or cytologically confirmed locally advanced or metastatic NSCLC measurable by Response Evaluation Criteria in Solid Tumors (RECIST) version 1.1 were enrolled in this study. Key eligibility requirements included age ≥70 years; no prior chemotherapy for metastatic disease; Eastern Cooperative Oncology Group (ECOG) PS 0 or 1; and adequate hematologic, renal, and liver function. Patients with active brain metastases or preexisting peripheral neuropathy (PN) grade ≥ 2 were excluded. Patients with a previously known epithelial growth factor receptor (*EGFR*) mutation or anaplastic lymphoma kinase (*ALK*) gene translocation must have had disease progression or proven intolerant to treatment with an EGFR inhibitor or ALK inhibitor, respectively.

This study was conducted in accordance with the Declaration of Helsinki and Good Clinical Practice Guidelines of the International Conference on Harmonisation. Informed consent was obtained from all patients before study entry. The trial is registered at ClinicalTrials.gov (NCT02151149).

### Randomization and Masking

The phase 4, randomized, open-label, multicenter ABOUND.70+ trial was conducted at 55 sites in the United States. Patients were randomized 1:1 to receive *nab*-paclitaxel 100 mg/m^2^ intravenously on days 1, 8, and 15 plus carboplatin area under the curve (AUC) 6 mg•min/mL intravenously on day 1 every 3 weeks (21d arm) or the same *nab*-paclitaxel/carboplatin doses every 3 weeks followed by a 1-week break before the start of the next cycle (21d + break arm; 28d) (Figure S1 in Supplementary Material). A permuted-block method was employed for randomization and carried out centrally using an interactive response technology system. Randomization was stratified by ECOG PS (0 vs 1) and histology (squamous vs nonsquamous). Treatment could continue in the absence of disease progression, unacceptable toxicity, or withdrawal of consent.

### Study Endpoints and Assessments

The primary endpoint was a composite safety endpoint and included the percentage of patients with either treatment-emergent grade ≥ 2 PN or treatment-emergent grade ≥ 3 myelosuppression (assessing for neutropenia, anemia, and thrombocytopenia based on National Cancer Institute Common Terminology Criteria for Adverse Events version 4.0). Secondary endpoints included safety, PFS, OS, and overall response rate (ORR). PFS and ORR were based on investigator’s assessments of data from computed tomography scans and RECIST 1.1 guidelines. PFS was defined as the time from randomization to disease progression or death from any cause in the absence of documented progression. OS was defined as the time from randomization to death from any cause. ORR was defined as the percentage of patients who had a radiological complete or partial response per RECIST version 1.1 with confirmation by radiological assessment ≥28 days later. Change in patient-reported QoL during the study was a prespecified exploratory endpoint. Safety was assessed in all patients who received at least one dose of study drug and included type, frequency, and severity of adverse events; discontinuation rate; dose intensity administered; and incidence of dose reductions and dose delays. PN (sensory or motor) was assessed by the investigator at screening, on days 1, 8, and 15 of each treatment cycle; at the end-of-treatment and 28-day follow-up visits; and at any time during the study as clinically indicated. Myelosuppression was assessed based on laboratory values for absolute neutrophil count, hemoglobin, and platelet count, which were collected at screening; on days 1, 8, and 15 of each cycle; at the end-of-treatment and 28-day follow-up visits; and as clinically indicated. Adverse events were classified by the Medical Dictionary for Regulatory Activities (MedDRA), and severity was assessed according to the National Cancer Institute Common Terminology Criteria for Adverse Events version 4.0; PN was defined by standardized MedDRA queries. Clinical laboratory data were collected, and dose reductions, delays, and discontinuations were implemented according to protocol-defined criteria (Table S1 in Supplementary Material). Efficacy was evaluated in the intent-to-treat population, which included all randomized patients. Tumors were assessed by computed tomography every 42 days starting from day 1 of cycle 1 until treatment discontinuation or withdrawal from study. Patients with available QoL data from baseline and at least one post-baseline visit were included in QoL analyses. The Lung Cancer Symptom Scale (LCSS) and the EuroQol 5D-5L (EQ-5D-5L) questionnaires were used to measure QoL, which was assessed on day 1 of each cycle.

### Statistical Analyses

The primary objective was to evaluate the impact of the two regimens on safety. Sample size was justified on the basis of the primary endpoint, a composite of the percentage of patients with relevant, treatment-emergent grade ≥ 2 PN or treatment-emergent grade ≥ 3 myelosuppression. The percentage of patients meeting at least one of the two criteria in the composite endpoint was summarized by each treatment arm, and the rate ratio (i.e., ratio of the percentages) was used to compare the 21d and 21d + break arms; the 21d regimen was the reference treatment (i.e., the rate in the denominator of the ratio). Ninety-five percent CIs were constructed for the rate ratio using the variance from a stratified Mantel–Haenszel estimator (7). Stratification variables included ECOG PS and histology. A stratified Mantel–Haenszel χ^2^ test was also used to assess a treatment effect for this safety endpoint.

The study was designed to detect a 16% difference between treatment arms with respect to the primary endpoint, with 80% power and a type I error of 5% (two sided), assuming the percentage of patients with grade ≥ 2 PN or grade ≥ 3 myelosuppression was 73% for the 21d arm. Therefore, approximately 284 patients were planned for 1:1 randomization to the 21d and 21d + break arms, with the expectation that approximately 278 patients (139 per group) would receive at least one dose of study drug and would be included in the treated population for analysis of the primary endpoint.

After approximately 120 treated patients completed 4 months of treatment or discontinued from the study, a prespecified interim analysis of the primary endpoint was performed, with the option of halting the study early if the difference in the primary endpoint between the two arms did not exceed prespecified criteria for futility (21d + break arm − 21d arm ≥ −0.2%).

Plots of Kaplan–Meier product-limit estimates were used to summarize the PFS and OS curves. Differences in the underlying PFS and OS curves were assessed using a log-rank test stratified by ECOG PS and histology. HRs and their CIs were estimated using a Cox proportional hazards model stratified by ECOG PS and histology. The comparison of response rates between treatment arms was assessed in the same manner as the primary endpoint. *P* values for the secondary endpoints are provided as a summary statistic to help identify any potential treatment effects. For statistical purposes, all QoL scales were aligned so that a positive change from baseline indicated improvement. Changes from baseline in LCSS, EQ-5D-5L, and visual analogue scale (VAS) items were described by descriptive statistics.

## Results

### Patients

**Figure 1:**
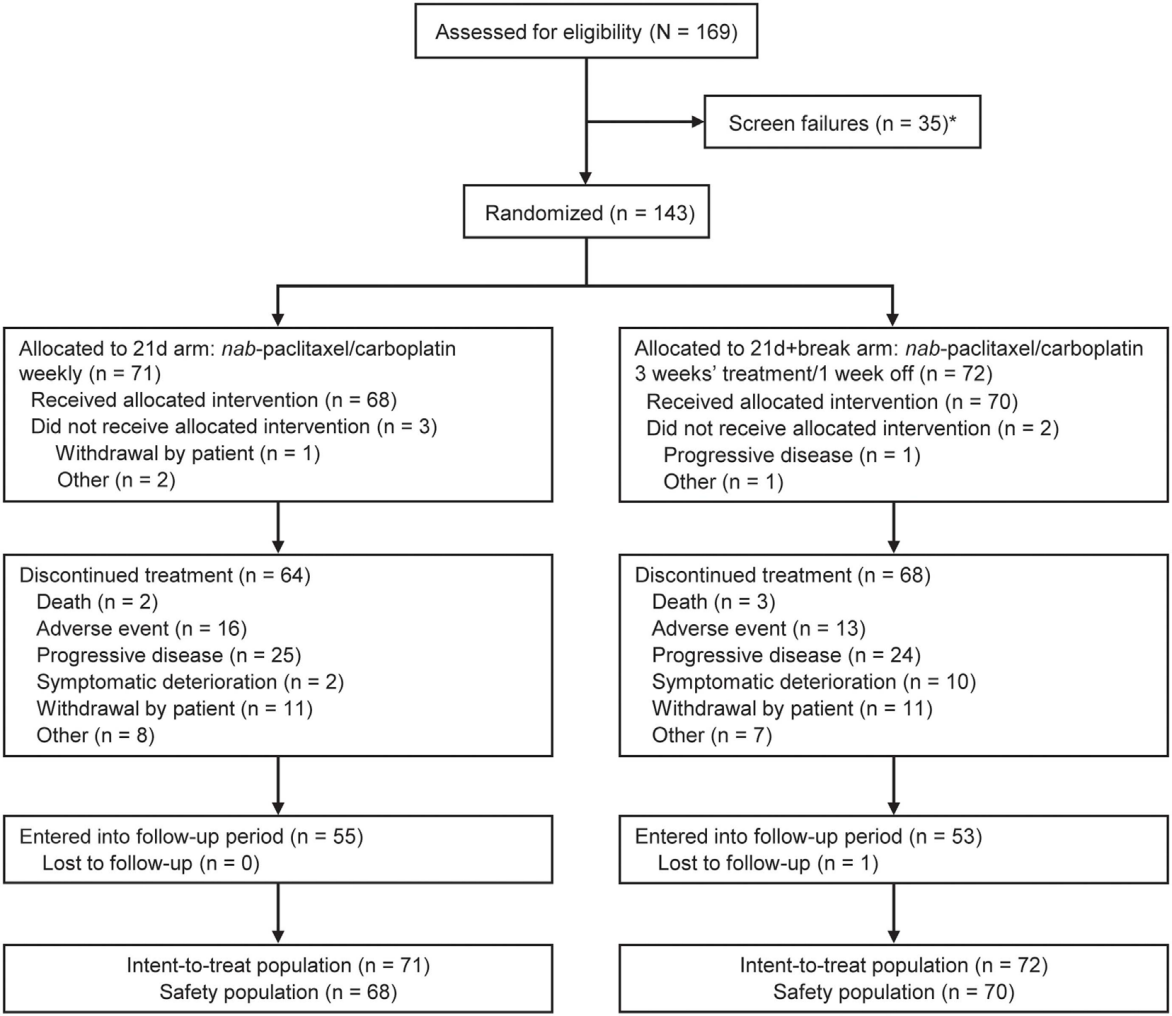
CONSORT diagram for ABOUND.70+. *A patient could have been screened more than once but was only counted once in total screened.

In total, 169 patients were screened for inclusion into the study; there were 35 screen failures; however, patients may have been screened more than once (Figure 1). At data cutoff (November 20, 2016), 143 patients had been randomized: 71 to the 21d arm and 72 to the 21d + break arm, which constituted the intent-to-treat population. Five patients (3 in the 21d arm and 2 in the 21d + break arm) did not receive treatment; 68 patients in the 21d arm and 70 patients in the 21d + break arm constituted the treated population, which was evaluated for safety. Baseline characteristics are summarized in Table 1. Overall, the distribution of patients among age categories was well balanced—37.1% were aged 70–74 years, 33.6% were 75–79 years, and 25.2% were 80–84 years. The median age in the 21d and 21d + break arms was 76.0 and 75.0 years, respectively, and most patients had an ECOG PS of 1 (70.4 vs 72.2%). In both arms, all patients had at least one comorbidity and reported taking at least one concomitant medication (Tables S2 and S3 in Supplementary Material). The percentage of patients with squamous histology was similar between the 21d and 21d + break arms (38.0 vs 38.9%), and most patients had stage IV disease (84.5 vs 81.9%). The incidence of physician-assessed PN at baseline was balanced between the 21d and 21d + break arms: 76.1 vs 79.2% had no PN at baseline, while 19.7 vs 18.1% had grade 1 and 1.4 vs 0% had grade 2. The 21d arm had a higher percentage of white patients (90.1 vs 79.2%) and patients aged 75–79 years (42.3 vs 25.0%), while the 21d + break arm had a higher percentage of patients aged 70–74 years (28.2 vs 45.8%) and black or African-American (4.2 vs 9.7%) and Asian patients (0 vs 4.2%).

**Table 1:** Baseline characteristics.

Patient characteristics	21d Arm (*n* = 71)	21d + Break Arm (*n* = 72)
Age, median, years (range)	76.0 (70.0–87.0)	75.0 (70.0–93.0)
70–74 years, *n* (%)	20 (28.2)	33 (45.8)
75–79 years, *n* (%)	30 (42.3)	18 (25.0)
≥80 years, *n* (%)	21 (29.6)	21 (29.2)
Sex, *n* (%)		
Male	41 (57.7)	40 (55.6)
Female	30 (42.3)	32 (44.4)
Race, *n* (%)		
White	64 (90.1)	57 (79.2)
Black or African-American	3 (4.2)	7 (9.7)
Asian	0	3 (4.2)
American Indian or Alaskan Native	0	1 (1.4)
Native Hawaiian or other Pacific Islander	0	1 (1.4)
Other	1 (1.4)	1 (1.4)
Unknown	3 (4.2)	2 (2.8)
Disease stage, *n* (%)		
IIIA	4 (5.6)	5 (6.9)
IIIB	6 (8.5)	8 (11.1)
IV	60 (84.5)	59 (81.9)
Missing	1 (1.4)	0
Histology, *n* (%)		
Nonsquamous	44 (62.0)	44 (61.1)
Squamous	27 (38.0)	28 (38.9)
ECOG PS, *n* (%)		
0	21 (29.6)	20 (27.8)
1	50 (70.4)	52 (72.2)
Physician assessment of PN at baseline, *n* (%)		
No PN	54 (76.1)	57 (79.2)
Grade 1	14 (19.7)	13 (18.1)
Grade 2	1 (1.4)	0
Data missing	2 (2.8)	2 (2.8)

*ECOG PS, Eastern Cooperative Oncology Group performance status; PN, peripheral neuropathy*.

### Primary Endpoint

At a prespecified non-binding interim evaluation, the scientific steering committee determined that the protocol futility criterion (primary endpoint treatment difference of 21d + break arm − 21d arm ≥ −0.2%) had been met; no difference was observed between treatment arms with respect to the primary endpoint. A decision was thus made to stop enrollment early.

A total of 68 patients in the 21d arm and 70 patients in 21d + break arm (safety population) were evaluated for safety (including the primary endpoint). At final analysis, the percentages of patients with either grade ≥ 2 PN or grade ≥ 3 myelosuppression adverse events were 76.5 and 77.1% for the 21d and 21d + break arms, respectively [risk ratio 1.01, 95% CI 0.84–1.21; *P* = 0.9258 (Table 2)]. The percentages of patients with grade ≥ 2 PN (36.8 vs 35.7%) and grade ≥ 3 myelosuppression (70.6 vs 64.3%) were comparable across treatment arms. In a *post hoc* analysis of the primary endpoint components, the time to onset of grade ≥ 2 PN and grade ≥ 3 myelosuppression was analyzed by the Kaplan–Meier method, with differences between arms compared using the Fleming-Harrington weighted log-rank test. The median time to first onset of grade ≥ 2 PN was shorter in the 21d arm than the 21d + break arm (5.3 vs 9.0 months; *P* = 0.0329) (Figure S2A in Supplementary Material). The median time to first onset of grade ≥ 3 myelosuppression was also shorter in the 21d arm than the 21d + break arm (1.1 vs 2.3 months; *P* = 0.0151) (Figure S2B in Supplementary Material).

**Table 2:** Primary endpoint.

Events, *n* (%)	21d Arm (*n* = 68)	21d + Break Arm (*n* = 70)
Patients with either grade ≥ 2 PN or grade ≥ 3 myelosuppression	52 (76.5)	54 (77.1)
95% CI	(64.6–85.9)	(65.6–86.3)
Risk ratio (95% CI)	1.01 (0.84–1.21)	
*P* value	0.9258	
Grade ≥ 2 PN	25 (36.8)	25 (35.7)
Grade ≥ 3 myelosuppression	48 (70.6)	45 (64.3)
Neutropenia	39 (57.4)	39 (55.7)
Anemia	14 (20.6)	17 (24.3)
Thrombocytopenia	17 (25.0)	12 (17.1)

PN, peripheral neuropathy.

### Treatment Exposure

Treatment exposure is shown in Table 3. *nab*-Paclitaxel dose intensity was higher in the 21d arm vs the 21d + break arm (62.0 vs 54.2 mg/m^2^/week), which was consistent with the protocol-specified dosing schedule. Median *nab*-paclitaxel cumulative dose was lower in the 21d arm than in 21d + break arm (875.0 vs 1287.5 mg/m^2^), as were median percentage of protocol dose (62.0 vs 72.2%) and median number of treatment cycles administered (4.0 vs 5.5); the median treatment duration was also shorter in the 21d arm than in 21d + break arm (3.0 vs 5.2 months). The percentage of patients with a *nab*-paclitaxel dose reduction/delay was higher in the 21d arm, and reductions from maximum *nab*-paclitaxel dose (100 mg/m^2^) occurred during earlier cycles in this arm. In the 21d and 21d + break arms, 64 (90.1%) and 68 (94.4%) patients discontinued treatment before the data cutoff due to progressive disease (35.2 vs 33.3%), adverse event (22.5 vs 18.1%), withdrawal by patient (15.5 vs 15.3%), symptomatic deterioration (2.8 vs 13.9%), or death (2.8 vs 4.2%).

**Table 3:** Treatment exposure^a^ and dose modifications.

Parameters	21d Arm (*n* = 68)	21d + Break Arm (*n* = 70)
Median dose intensity		
*nab*-Paclitaxel, mg/m^2^/week	62.0	54.2
Carboplatin, AUC/week	1.46	1.24
Median cumulative dose		
*nab*-Paclitaxel, mg/m^2^	875.0	1287.5
Carboplatin, AUC	20.3	29.3
Median percentage of per-protocol dose		
*nab*-Paclitaxel	62.0	72.2
Carboplatin	73.0	82.8
Treatment duration, median, months	3.0	5.2
Median number of cycles administered	4.0	5.5
Patients with ≥1 dose not administered, *n* (%)		
*nab*-Paclitaxel	55 (80.9)	57 (81.4)
Carboplatin	9 (13.2)	7 (10.0)
Patients with ≥1 dose delay, *n* (%)		
*nab*-Paclitaxel	40 (58.8)	34 (48.6)
Carboplatin	36 (52.9)	31 (44.3)
Patients with ≥1 dose reduction, *n* (%)		
*nab*-Paclitaxel	44 (64.7)	41 (58.6)
Carboplatin	39 (57.4)	41 (58.6)

*AUC, area under the curve*.

*^a^Statistical comparisons were not performed for treatment exposure data*.

### Safety

In the 21d arm vs the 21d + break arm, 100 vs 97.1% of patients had at least one treatment-emergent adverse event and 95.6 vs 94.3% had at least one treatment-related adverse event. The most common treatment-emergent adverse events in both arms (≥40%) were anemia, fatigue, peripheral sensory neuropathy, neutropenia, nausea, diarrhea, and alopecia. The occurrence of grade ≥ 3 adverse events was similar between arms (88.2 vs 85.7%), but more patients in the 21d + break arm (44.3%) experienced a serious adverse event compared with patients in the 21d arm (33.8%). One patient in the 21d arm and three in the 21d + break arm had fatal treatment-emergent adverse events; none were considered treatment related.

Overall, grade ≥ 3 adverse events of special interest were mainly hematologic, followed by gastrointestinal events and PN (Table 4). Among adverse events of special interest, the incidences of all-grade neutropenia (73.5 vs 64.3%), anemia (67.6 vs 57.1%), thrombocytopenia (54.4 vs 34.3%), and PN (60.3 vs 47.1%) were higher (≥ 5% difference) in the 21d arm, while vomiting (22.1 vs 31.4%) was more common in the 21d + break arm.

**Table 4:** Adverse events of special interest.

Adverse events, ***n*** (%)	21d Arm (*n* = 68)		21d + Break Arm (*n* = 70)	
	All grade	Grade ≥ 3	All grade	Grade ≥ 3
General myelosuppression				
Neutropenia	50 (73.5)	37 (54.4)	45 (64.3)	37 (52.9)
Anemia	46 (67.6)	15 (22.1)	40 (57.1)	16 (22.9)
Thrombocytopenia	37 (54.4)	17 (25.0)	24 (34.3)	12 (17.1)
Peripheral neuropathy	41 (60.3)	9 (13.2)	33 (47.1)	12 (17.1)
Gastrointestinal events				
Diarrhea	30 (44.1)	11 (16.2)	28 (40.0)	4 (5.7)
Nausea	33 (48.5)	3 (4.4)	36 (51.4)	3 (4.3)
Vomiting	15 (22.1)	2 (2.9)	22 (31.4)	3 (4.3)
Arthralgia	5 (7.4)	0	10 (14.3)	2 (2.9)

The proportion of patients with grade ≥ 3 PN in the 21d arm vs the 21d + break arm was 13.2 vs 17.1%. Among patients who developed grade ≥ 3 PN, the median time to onset was not estimable vs 11.3 months in the 21d and 21d + break arms, respectively. Improvement by at least one grade (from grade ≥ 3 PN) was observed in 5 of 9 patients in the 21d arm and 8 of 12 patients in the 21d + break arm, and the median time to improvement by at least one grade was 4.6 vs 7.6 months. Peripheral sensory neuropathy was the most common adverse event leading to study drug withdrawal in either arm (5.9% in the 21d arm and 8.6% in the 21d + break arm).

### Efficacy

**Figure 2:**
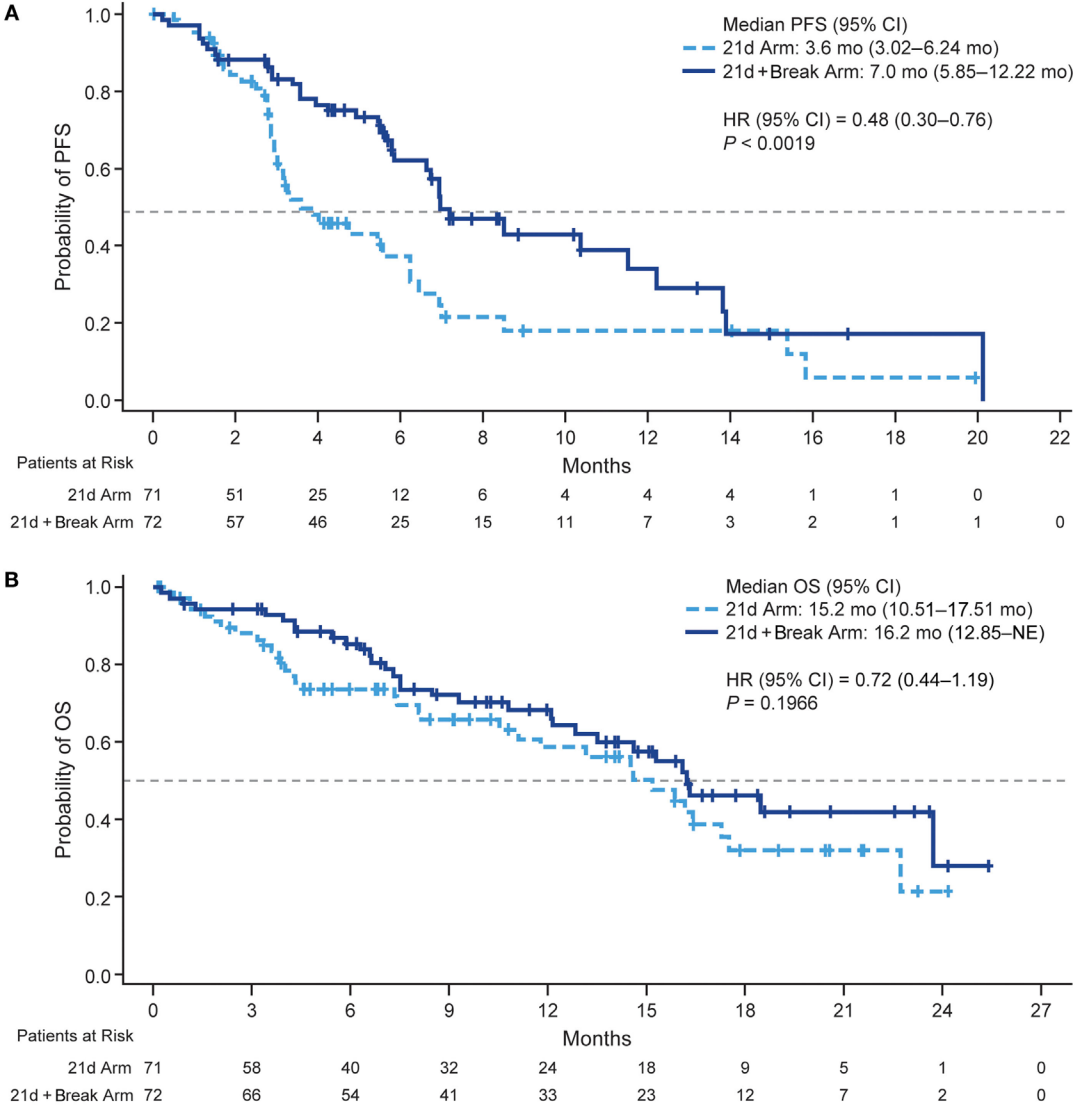
Kaplan–Meier plots of PFS and OS. **(A)** PFS. **(B)** OS. Abbreviations: HR, hazard ratio; NE, not estimable; PFS, progression-free survival; OS, overall survival.

Median PFS was 3.6 and 7.0 months (HR 0.48, 95% CI 0.30–0.76; *P* < 0.0019) (Figure 2A) in the 21d and 21d + break arms, respectively, which corresponded to a 52% reduction in risk of death or progression in patients in the 21d + break arm. There were 34 deaths (47.9%) in the 21d arm and 31 (43.1%) in the 21d + break arm. The median OS was 15.2 and 16.2 months (HR 0.72, 95% CI 0.44–1.19; *P* = 0.1966) (Figure 2B); median follow-up times were 15.8 and 15.9 months, respectively. One-year OS rates were 59.0% (95% CI 44.0–70.0%) and 68.0% (95% CI 55.0–78.0%), and the 2-year OS rates were 22.0% (95% CI 6.0–43.0%) and 28.0% (95% CI 8.0–53.0%) in the 21d and 21d + break arms, respectively. In an updated analysis with 2-year survival follow-up, median OS was 14.5 and 15.0 months (HR 0.82, 95% CI 0.54–1.25; *P* = 0.3537) in the 21d and 21d + break arms, respectively. Analysis of OS by subgroups showed no differences between treatment arms by age, ECOG PS, or sex; a potential OS trend was observed in favor of the 21d + break arm in patients with nonsquamous cell carcinoma histology (HR 0.64, 95% CI 0.37–1.11) (Figure S3 in Supplementary Material). To assess the impact of subsequent therapy on OS, a sensitivity analysis was performed. When patients were censored at initiation of subsequent systemic anticancer therapy, median OS was 11.10 and 16.10 months (HR 0.53, 95% CI 0.30–0.95; *P* = 0.0291) in the 21d and 21d + break arms, respectively (Figure S4 Supplementary Material).

**Figure 3:**
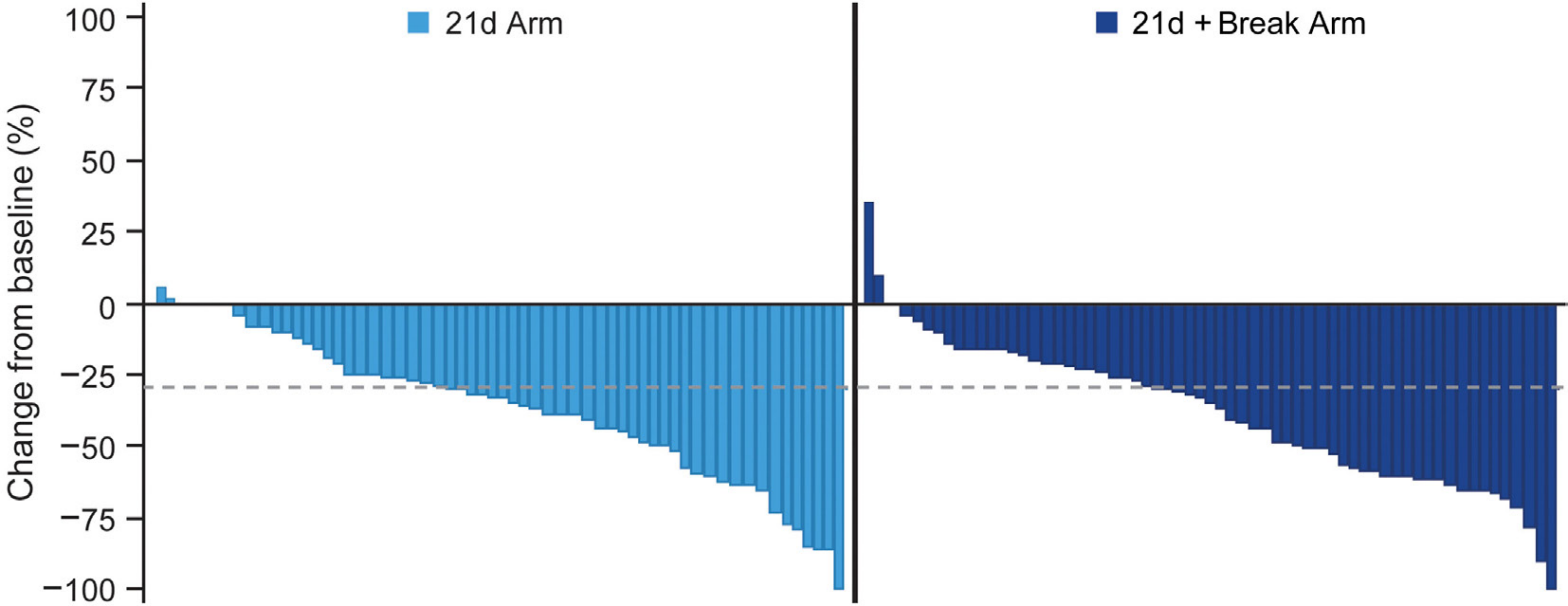
Waterfall plot of best percent change from baseline in total length of longest diameters of target lesions.

The confirmed ORRs in the 21d and 21d + break arms were 23.9 and 40.3% (risk ratio 1.68, 95% CI 1.02–2.78; *P* = 0.0376), respectively (Table 5). The majority of patients in both arms had tumor shrinkage. In the 21d and 21d + break arms, 46.5 and 48.6% of patients had a best percentage target lesion decrease from baseline of ≥30%, respectively (Figure 3). The median best percentage change from baseline of target lesions was −33.33 and −33.96%, respectively. In each arm, one patient achieved a confirmed complete response. In the 21d arm, an 80-year-old patient who received a total of six treatment cycles achieved a complete response at cycle 6. Dose modifications for this patient included *nab*-paclitaxel dose reductions at cycle 1 day 8 to 75 mg/m^2^ and again at cycle 3 day 15 to 50 mg/m^2^ and carboplatin dose reductions at cycle 2 day 1 to AUC 4.5 and again at cycle 4 day 1 to AUC 3. In the 21d + break arm, a 71-year-old patient who also received a total of six treatment cycles achieved complete response by cycle 2. Dose modifications for this patient included *na*b-paclitaxel dose reductions at cycle 3 day 1 to 75 mg/m^2^ and again at cycle 5 day 1 to 50 mg/m^2^ and carboplatin dose reductions to AUC 4.5 and AUC 3 at cycle 3 day 1 and cycle 5 day 1, respectively.

**Table 5:** Best response to treatment.

Response, *n* (%)	21d Arm (*n* = 71)	21d + Break Arm (*n* = 72)
Confirmed ORR	17 (23.9)	29 (40.3)
RR (95% CI)	1.68 (1.02–2.78)	
*P* value	0.0376	
Complete response	1 (1.4)	1 (1.4)
Partial response	16 (22.5)	28 (38.9)
Stable disease	37 (52.1)	31 (43.1)
Progressive disease	7 (9.9)	4 (5.6)
No post-baseline response data	10 (14.1)	8 (11.1)

*ORR, overall response rate; RR, risk ratio*.

### Quality of Life

**Figure 4:**
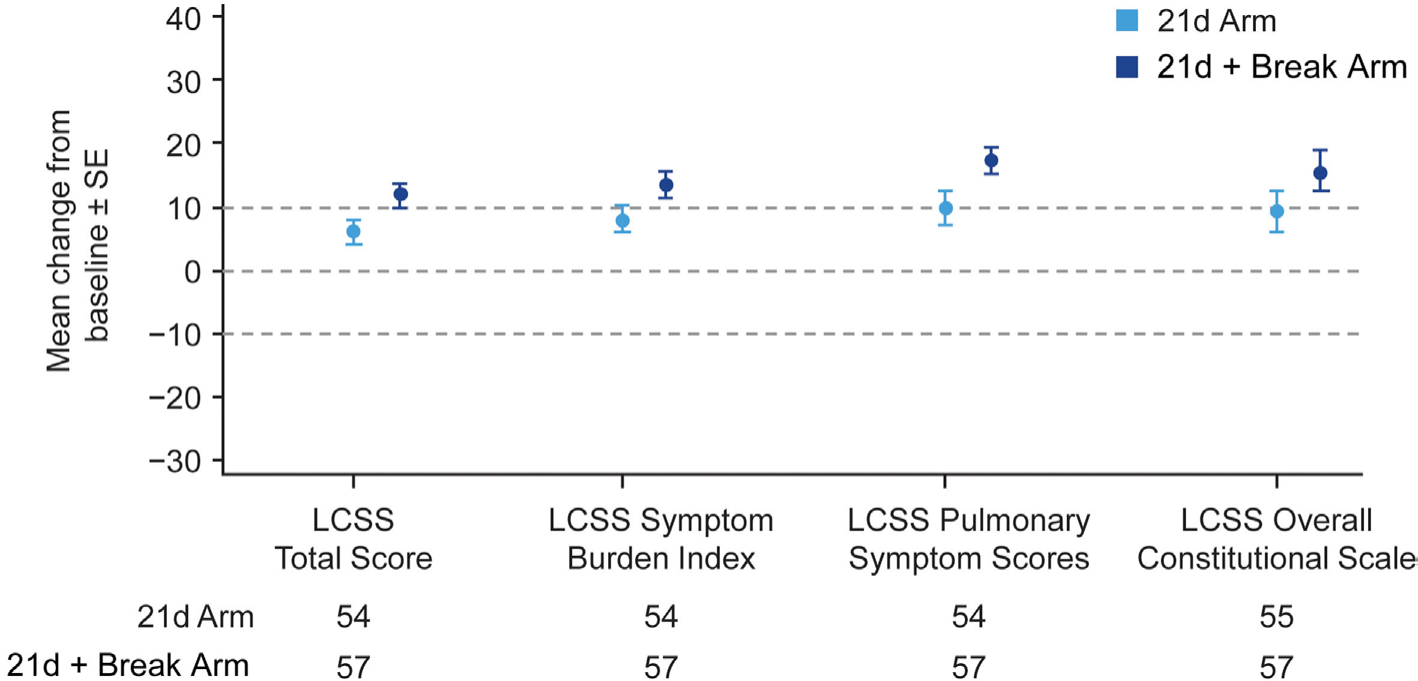
Mean maximum change from baseline in LCSS scores. Abbreviation: LCSS, Lung Cancer Symptom Scale. *Not all patients completed assessments in all categories.

**Figure 5:**
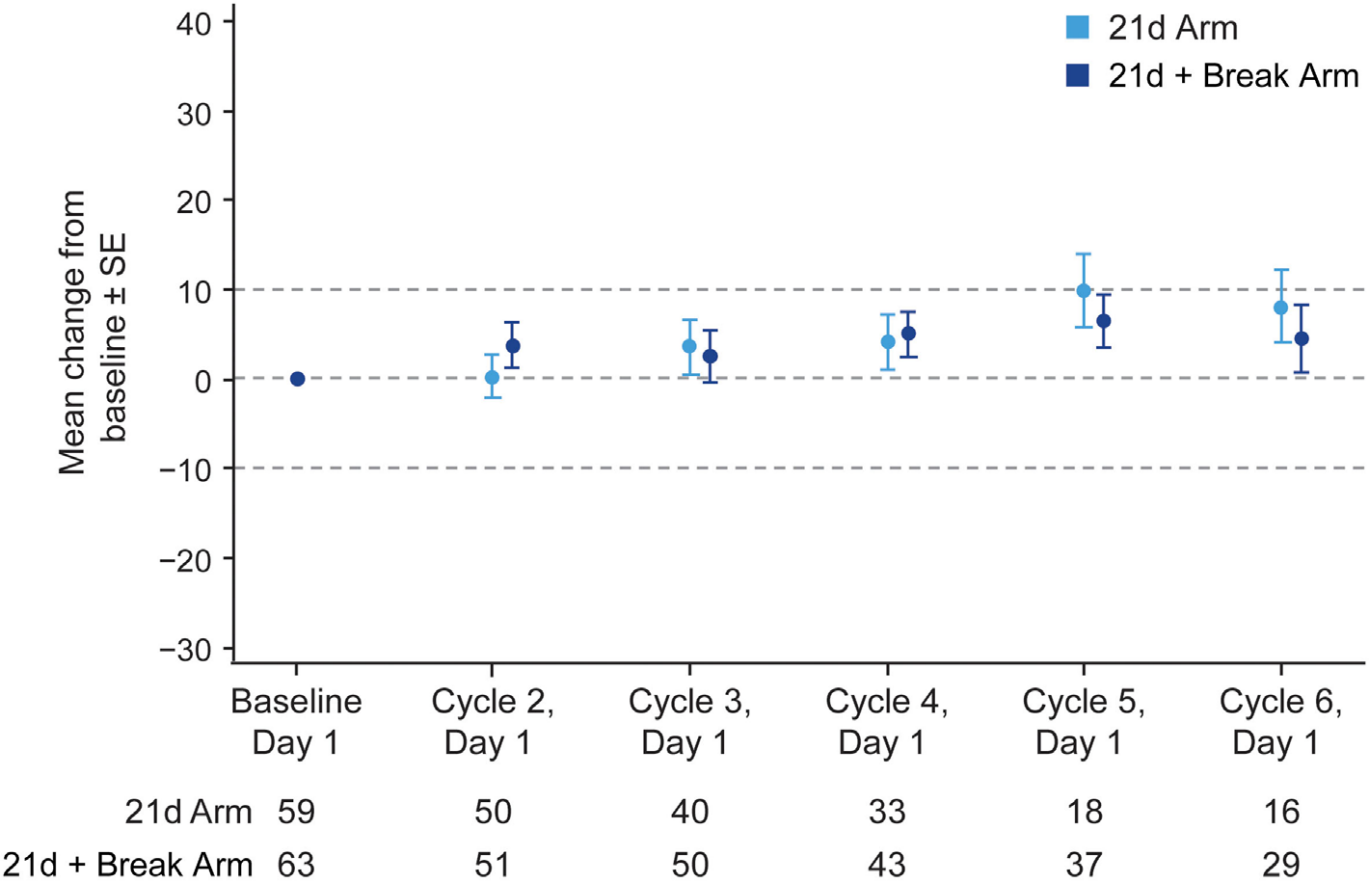
Change in LCSS pulmonary symptom score from baseline by treatment arm. Abbreviation: LCSS, Lung Cancer Symptom Scale. *Not all patients completed assessments at all the time points.

The majority of patients in the 21d and 21d + break arms (77.5 and 79.2%, respectively) had a baseline and at least one post-baseline QoL assessment. In general, patients reported improvements in the LCSS items of average total, average symptom burden index, pulmonary symptom (cough, shortness of breath, hemoptysis), and overall constitutional scale scores (Figure 4; Figure S5 Supplementary Material). Mean changes from baseline in LCSS pulmonary symptom scale scores were positive in both the 21d and 21d + break arms (Figure 5). In the LCSS item of cough, mean changes from baseline in the 21d and 21d + break arms were 19.8 and 15.4 mm (VAS) at the end of cycle 5. EQ-5D VAS scores indicated improvements from baseline QoL in both arms; the mean maximum improvements (at any point during treatment) for EQ-5D VAS was 11.6 and 12.9 points in the 21d and 21d + break arms, respectively.

## Discussion

Overall, results from the ABOUND.70+ trial support the findings of prior subset analyses on the safety and efficacy of *nab*-paclitaxel/carboplatin for first-line use in elderly patients with advanced NSCLC (5). Although the 1-week break between cycles did not reduce the overall percentage of patients with grade ≥ 2 PN or grade ≥ 3 myelosuppression (primary endpoint), patients who received a 1-week break between cycles realized clear benefits, including a longer treatment duration and greater cumulative dose that likely contributed to the longer PFS and higher confirmed ORR. Overall, there was no detriment to patient QoL in either arm; the data suggest that a 1-week break may have improved QoL outcomes. Adverse event profiles were generally similar between treatment arms, although patients who received a 1-week break had a later onset of grade ≥ 2 PN.

The adverse event profile of *nab*-paclitaxel/carboplatin in ABOUND.70+ was generally consistent with that observed in the phase 3 trial, with grade ≥ 3 adverse events mainly hematologic in nature; the rates of neutropenia, anemia, and thrombocytopenia were comparable to those of the overall treated population and the elderly subset from the phase 3 trial (5). Although grade ≥ 2 PN rates were higher in this study than those historically reported with *nab*-paclitaxel/carboplatin in patients with NSCLC, several factors may have contributed to these differences (5, 8), including a higher incidence of baseline PN and a more frequent schedule of PN assessment in this study than in the prior phase 3 trial (8). In addition, this study reported treatment-emergent PN (sensory and motor), whereas the phase 3 trial reported treatment-related sensory neuropathy (5, 9). Regional differences in the management of PN may have also played a role in the observed variances: ABOUND.70+ was a US-based study, while the phase 3 trial was global. Notably, similar rates of grade ≥ 2 PN were observed in both arms of ABOUND.70+, despite differences in treatment exposure.

Data from this study prospectively support the efficacy of *nab*-paclitaxel/carboplatin in elderly patients with NSCLC observed in the prior phase 3 study; a subgroup analysis of elderly patients from the phase 3 trial comparing *nab*-paclitaxel/carboplatin with paclitaxel/carboplatin demonstrated a median OS and PFS of 19.9 and 8.0 months, respectively, and an ORR of 34% (5). To date, the median OS values reported with *nab*-paclitaxel/carboplatin treatment of elderly patients in this study and in the elderly subset of the phase 3 trial are among the longest reported in elderly patients with advanced NSCLC. It should be noted that studies of pemetrexed/carboplatin have indicated that a carboplatin dose of AUC 5 may be more tolerable in this population (10, 11). However, other large studies of taxane-based doublets have routinely examined carboplatin AUC 6 in elderly patients (4, 5, 9). Notably, as demonstrated in the phase 3 trial, an AUC of 6 was well tolerated by the subset of elderly patients treated with both *nab*-paclitaxel/carboplatin and paclitaxel/carboplatin; the safety findings in this population were similar to those of the intent-to-treat population and those of patients <70 years (5, 9). Furthermore, when put in context of historical studies, the current study underscores the value of carboplatin-based doublet therapy, including *nab*-paclitaxel/carboplatin, as a standard of care for fit elderly patients (4, 12).

This study had some limitations that warrant acknowledgment. The full intended enrollment was not completed due to a decision to stop the study for futility (no advantage for the 21d + break arm observed with respect to the primary endpoint) based on an interim analysis, resulting in patient accrual numbers that were lower than those originally specified in the protocol. Final analysis of the primary endpoint was consistent with the interim analysis. The statistical inferences (i.e., *P* values and 95% CIs) associated with PFS, OS, and ORR should be interpreted with caution because they were calculated without control for either type I or II errors in a study that was stopped early for futility of a safety endpoint. In addition, because the primary focus of the trial was safety (primary endpoint), the sample size was calculated for the primary endpoint and was underpowered for the efficacy endpoints. Furthermore, OS may have been influenced by subsequent lines of therapy, as indicated by the sensitivity analysis. Patients in the 21d + break arm had longer disease control, which may have been due to the effect of greater treatment exposure. Criteria for inclusion in the study included ECOG PS ≤ 1; apart from baseline comorbidities and concomitant medications, there were no data collected to further characterize patient fitness and comorbidities. In addition, the relatively small population in this study precludes further analyses grouped by existing fitness categories; an analysis of a pooled patient population is underway and will be reported at a future date. QoL was measured only during the treatment phase and not during survival follow-up; therefore, the impact of each respective regimen on post-treatment QoL could not be assessed. Finally, although much of the importance of the data lie in supporting the merits of doublet therapy and *nab*-paclitaxel/carboplatin treatment for the elderly, this study did not directly compare outcomes with *nab*-paclitaxel/carboplatin to those with weekly paclitaxel/carboplatin. Nonetheless, it should be noted that this was addressed by the phase 3 study, and the general purpose of the current study was to understand how schedule could impact the tolerability profile of *nab*-paclitaxel/carboplatin in elderly patients with advanced NSCLC. One could envision a future study comparing carboplatin and weekly *nab*-paclitaxel with carboplatin and weekly solvent-based paclitaxel in elderly patients with advanced NSCLC.

In conclusion, overall results from the ABOUND.70+ trial support the prior findings of the subset analysis of the phase 3 trial that studied the safety and efficacy of *nab*-paclitaxel/carboplatin in patients ≥70 years with advanced NSCLC. The 1-week break did not reduce the percentage of patients meeting at least one of the two criteria in the composite endpoint (either grade ≥ 2 PN or grade ≥ 3 myelosuppression) compared with the continuous weekly schedule. OS was also similar between the two arms; however, a significant improvement in PFS and ORR was observed in patients treated with the 1-week break, which may have been the result of increased treatment exposure and delayed onset to grade ≥ 2 PN afforded by the scheduled 1-week break. In addition, efficacy outcomes in both arms generally exceeded those observed in key historical trials. Taken together, these results expand the body of knowledge for treating elderly patients with NSCLC and further support *nab*-paclitaxel/carboplatin as a standard of care in this vulnerable patient population.

## ABOUND.70+ Investigators

**Ajeet Gajra**, SUNY Upstate Medical University, Syracuse, NY, United States. **Andrei Dobrescu**, Regional Cancer Care Associates LLC Somerset Division, Somerville, NJ, United States. **Bohdan E. Halibey**, Regional Cancer Care Associates LLC—Sparta Division, Sparta, NJ, United States. **Corey Langer**, Abramson Cancer Center of the University of Pennsylvania, Philadelphia, PA, United States. **Daniel Haggstrom**, Levine Cancer Institute, Charlotte, NC, United States. **David A. Smith**, Northwest Cancer Specialists, P.C., Vancouver, WA, United States. **Eric Anderson**, 1130 N.W. 22nd Avenue, Portland, OR, United States. **Eugene H. Paschold**, Novant Health Oncology Specialists, Winston Salem, NC, United States. **Haiying Cheng**, Montefiore-Einstein Center for Cancer Care, Bronx, NY, United States. **Haythem Ali**, Henry Ford Health System, Detroit, MI, United States. **Hossein Borghaei**, Fox Chase Cancer Center, Philadelphia, PA, United States. **Jared Weiss**, University of North Carolina at Chapel Hill, Chapel Hill, NC, United States. **Jawad Elias Francis** and **Ayla Ahmed Kessler**, St Elizabeth Youngstown Hospital, Youngstown, OH, United States. **Jen C. Wang**, The Brookdale University Hospital and Medical Center, Brooklyn, NY, United States. **Jonathan Wade Goldman**, 10945 Le Conte Avenue, Los Angeles, CA, United States. **Jose E. Najera** and **Nadim F. Nimeh**, Cancer Centers of Southwest Oklahoma, Lawton, OK, United States. **Joseph Rosales**, Virginia Mason Medical Center, Seattle, WA, United States. **Kartik Konduri**, Texas Oncology-Baylor Charles A. Sammons Cancer Center, Dallas, TX, United States. **Konstantin H. Dragnev**, Dartmouth-Hitchcock Medical Center, Lebanon, NH, United States. **Leonardo Forero**, Texas Oncology-Amarillo, Amarillo, TX, United States. **Lynne A. Bui**, Global Cancer Research Institute (GCRI), Inc., Gilroy, CA, United States. **Marc R. Matrana**, Ochsner Clinic Foundation, New Orleans, LA, United States. **Matei P. Socoteanu**, Texas Oncology-Longview Cancer Center, Longview, TX, United States. **Maurice Willis**, University of Texas Medical Branch, Galveston, TX, United States. **Monika Joshi**, Penn State Milton S. Hershey Medical Center, Penn State Hershey Cancer Institute, Hershey, PA, United States. **Morton Coleman**, Morton Coleman, MD, New York, NY, United States. **Moses Sundar Raj**, Allegheny General Hospital, Pittsburgh, PA, United States. **Navkiranjit Gill**, Oncology Specialists, SC, Park Ridge, IL, United States. **Patricia M. Plezia** and **Manuel R. Modiano**, ACRC/Arizona Clinical Research Center, Inc., Tucson, AZ, United States. **R. Timothy Webb**, Genesis Cancer Center, Hot Springs, AZ, United States. **Rita Axelrod**, 1025 Walnut Street, Philadelphia, PA, United States. **Robert Andrew Dichmann**, Central Coast Medical Oncology Corporation, Santa Maria, CA, United States. **Robert M. Jotte**, Rocky Mountain Cancer Centers, Denver, CO, United States. **Ronald P. Harris**, Broome Oncology LLC, Johnson City, NY, United States. **Scott Anthony Sonnier** and **Vijay Patel**, Crescent City Research Consortium, LLC, Marrero, LA, United States. **Shaker R. Dakhil**, Cancer Center of Kansas, Wichita, KS, United States. **Tarek Mekhail**, Cancer Institute of Florida, Orlando, FL, United States. **Thomas Hensing**, NorthShore University HealthSystem-Evanston Hospital, Evanston, IL, United States. **Tony M. Samaha**, Reliant Medical Group, Inc., St. Vincent Hospital Cancer and Wellness Center, Worcester, MA, United States. **Vicky Lee** and **Kimberly McGregor**, Good Samaritan Hospital Corvallis—Samaritan Pastega Regional Cancer Center, Corvallis, OR, United States. **William Eyre Lawler**, St. Jude Hospital Yorba Linda DBA Joseph Heritage Healthcare, Fullerton, CA, United States. **William L. Skinner**, West KY Hematology & Oncology Group, PSC-DBA: Paducah Cancer & Blood Center, Paducah, KY, United States. **William T. DeRosa**, Summit Medical Group, Morristown, NJ, United States.

## Author Contributions

CL, EK, JG, and JW conceived of and designed the study. EK, EA, RJ, MM, DH, MS, DS, CD, JG, and JW contributed to data collection. CL, EK, DH, DS, CD, TB, JG, JW, and AS analyzed the data. CL, EK, RJ, MM, DH, KK, TB, TO, AS, KA, and JG interpreted the data. EK drafted the report. CL, EK, EA, RJ, MM, DH, MS, DS, CD, KK, TB, TO, AS, KA, JG, and JW revised the report critically. All the authors reviewed and approved the final version of the report to submit for publication; agreed to be accountable for all aspects of the work and to ensure that questions related to the accuracy or integrity of any part of the work are appropriately investigated and resolved.

## Conflict of Interest Statement

CL is a consultant/advisor at Celgene, the study sponsor. EK received grants from Celgene during the conduct of the study. EA, RJ, MM, DH, MS, CD, and KK have nothing to disclose. DS led trials at US Oncology during the conduct of the study and outside the submitted work. TB, TO, AS, and KA are employees of Celgene. JG received grants from Celgene during the conduct of the study. JW has received grants from Celgene, Astellas, Merck, AstraZeneca, GlaxoSmithKline/Novartis, and Pfizer and personal fees from Celgene, Biodesix, EMD Serono, AstraZeneca, Genentech, Pfizer, Biomarck, Eli Lilly, and Oncoplex outside the submitted work. The reviewer AO and the handling Editor declared their shared affiliation.
