# Supplementary material for: nab-Paclitaxel-Based Therapy in Underserved Patient Populations: The ABOUND.70+ Study in Elderly Patients With Advanced NSCLC
Source: Front Oncol. 2018 Jul 24;8:262. doi: 10.3389/fonc.2018.00262 (PMC6066531; doi:10.3389/fonc.2018.00262)
Supplement: Supplementary file 1 [file Presentation_1.PDF]

Supplemental Figure 1: Study design

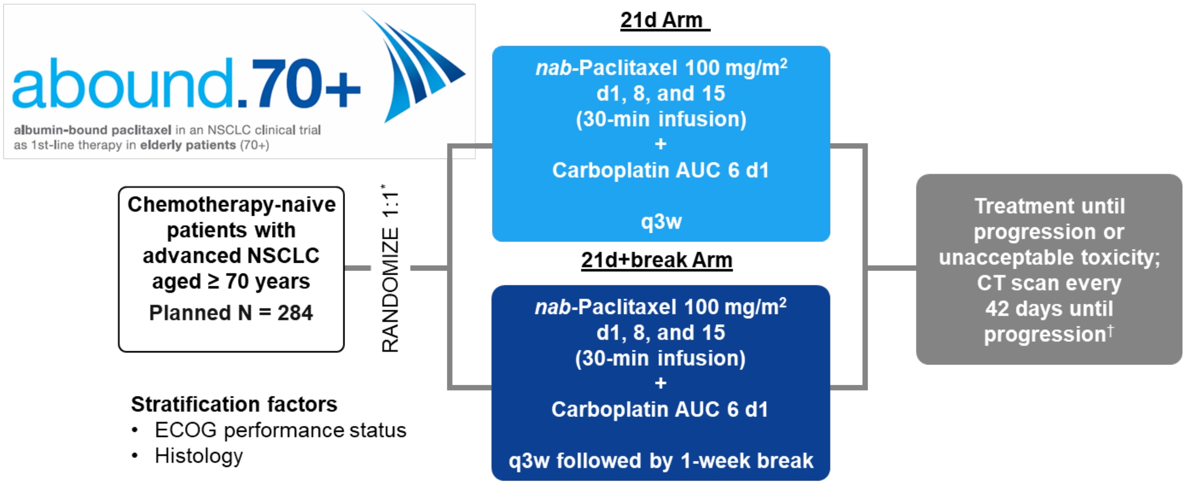

Primary endpoint: percentage of patients with either grade ≥ 2 peripheral neuropathy‡ or grade ≥ 3 myelosuppression§
Secondary endpoints: PFS, OS, ORR, safety

* After ≈ 120 patients completed 4 months of treatment or discontinued from the study, a pre-specified interim analysis of the primary safety endpoint was performed with the option of prematurely halting the study if the treatment difference between the two arms did not exceed pre-specified criteria.
† Follow-up period: 28-day follow-up visit (after treatment discontinuation) and thereafter approximately every 90 days for 6 months after last patient is randomised.
‡ Peripheral neuropathy events were identified from the clinical adverse event data set using Standardized MedDRA Queries.
§ Based on local laboratory values for absolute neutrophil count, platelet count, and haemoglobin.

AUC=area under the concentration curve. CT=computed tomography. ECOG=Eastern Cooperative Oncology Group. MedDRA=Medical Dictionary for Regulatory Activities. NSCLC=non-small cell lung cancer. ORR=overall response rate. OS=overall survival. PFS=progression-free survival. q3w=every 3 weeks.

Supplemental Figure 2: Kaplan-Meier plot of time to first onset of primary endpoint components **(A)** Grade ≥ 2 peripheral neuropathy. **(B)** Grade ≥ 3 myelosupression.

A.

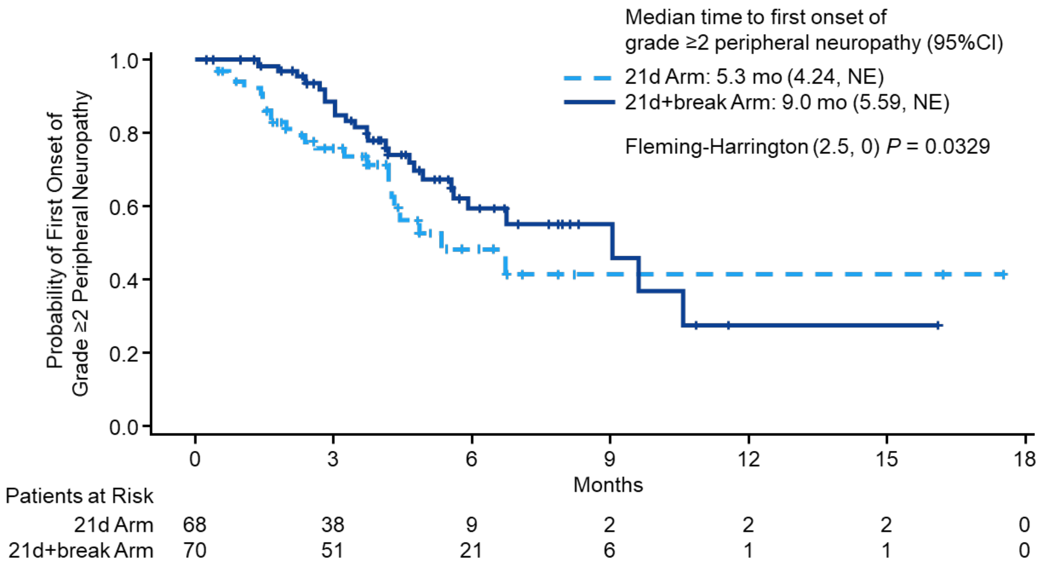

B.

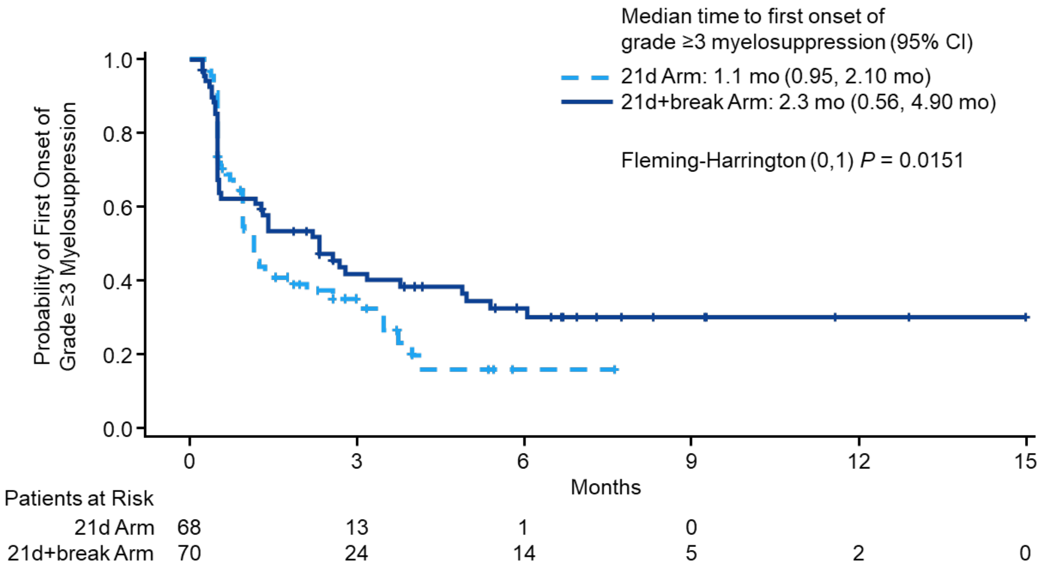

HR=hazard ratio. NE=not estimable.

Supplemental Figure 3: Forrest plot of overall survival by subgroup

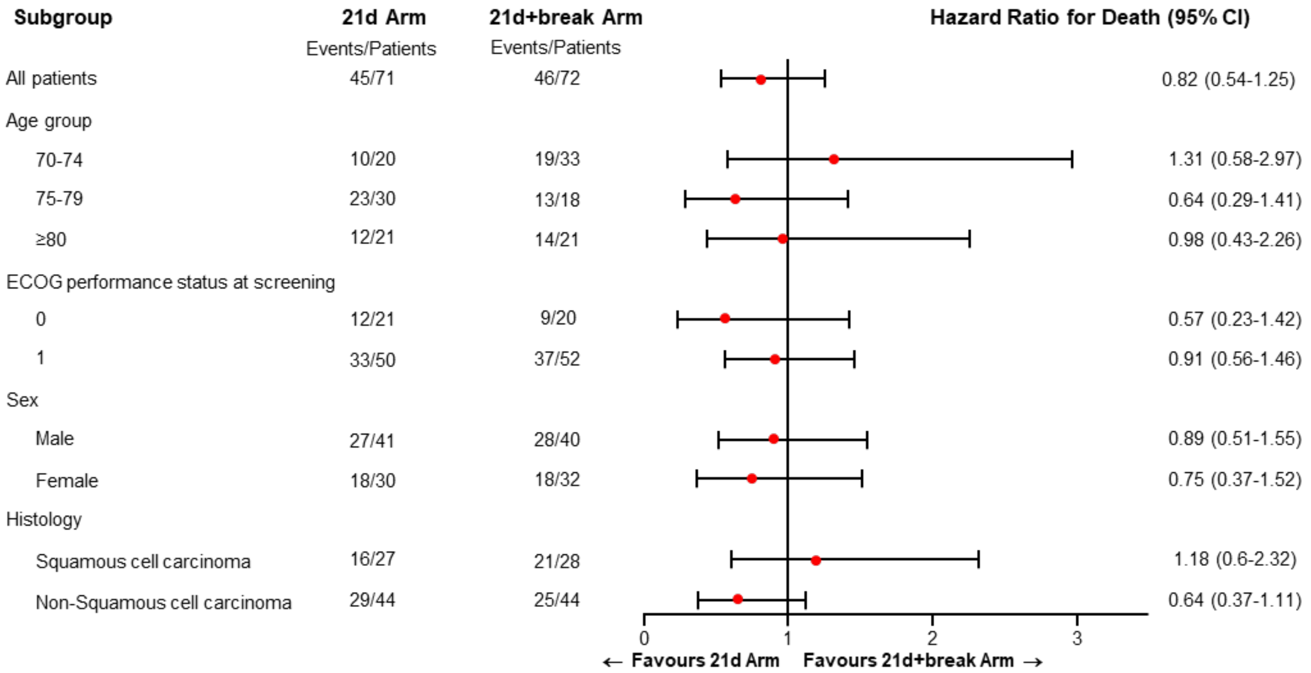

ECOG=Eastern Cooperative Oncology Group.

Supplemental Figure 4: Kaplan-Meier plot of overall survival censored at subsequent systemic anticancer treatment

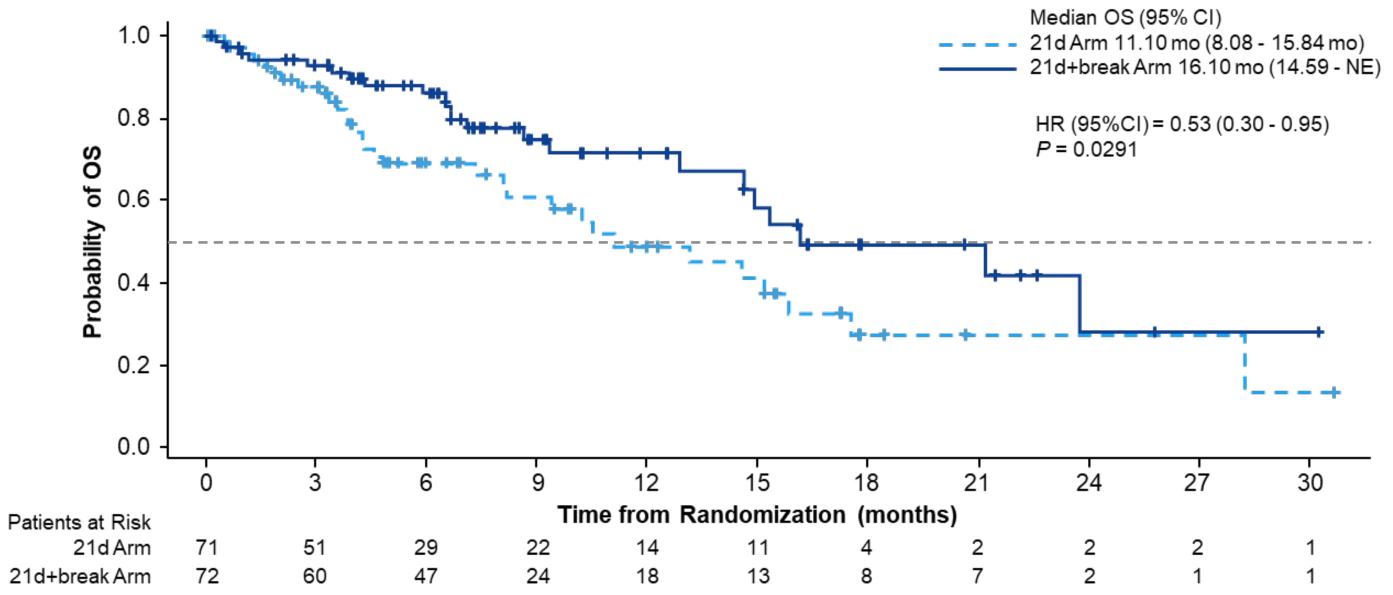

HR=hazard ratio. NE=not estimable.

Supplemental Figure 5: Change in LCSS scores from baseline by treatment arm

(A) LCSS total score. **(B)** LCSS symptom burden index. **(C)** Overall constitutional scale.

A.

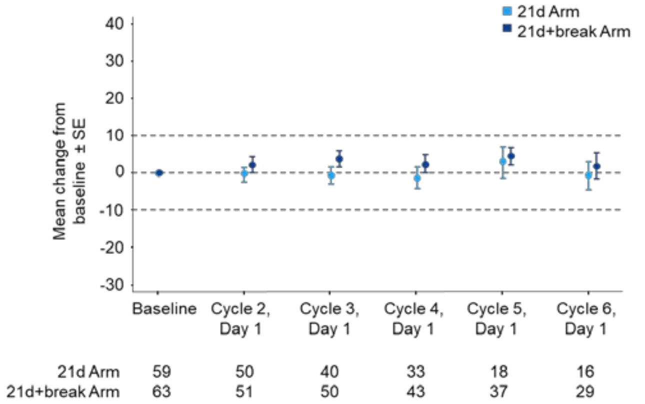

B.

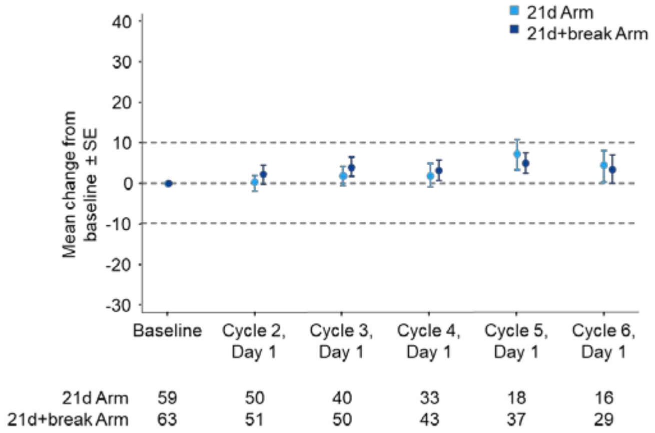

C.

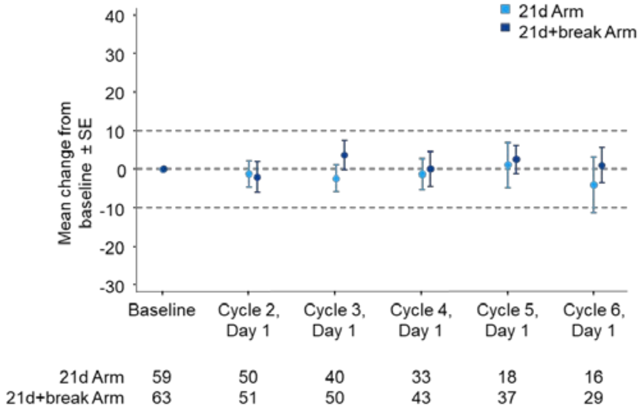

LCSS=Lung Cancer Symptom Scale
