# Supplementary material for: nab-Paclitaxel-Based Therapy in Underserved Patient Populations: The ABOUND.70+ Study in Elderly Patients With Advanced NSCLC
Source: Front Oncol. 2018 Jul 24;8:262. doi: 10.3389/fonc.2018.00262 (PMC6066531; doi:10.3389/fonc.2018.00262)
Supplement: Supplementary file 2 [file Table_1.docx]

***Supplemental Table 1*: Permanent dose reductions for hematologic and neurological toxicities and dosing on the study**

| **Dose modification during treatment** | | | |
| --- | --- | --- | --- |
| **Adverse drug reactions** | **Occurrence** | ***nab*-Paclitaxel dose (mg/m^2^)** | **Carboplatin dose (AUC mg•min/mL)** |
| Neutropenic fever (ANC < 500/mm^3^ with fever > 38°C)  ***or***  Delay of next cycle by > 7 days for ANC < 1500/mm^3^  ***or***  ANC < 500/mm^3^ for > 7 days | First | 75 | 4.5 |
|  | Second | 50 | 3.0 |
|  | Third | Discontinue treatment* | |
| Platelet count < 50,000/mm^3^ | First | 75 | 4.5 |
|  | Second | Discontinue treatment* | |
| Sensory neuropathy grade 3 or 4 | First | 75 | 4.5 |
|  | Second | 50 | 3.0 |
|  | Third | Discontinue treatment* | |
| Grade 2 or 3 cutaneous toxicity  Grade 3 diarrhea  Grade 3 mucositis  Any other grade 3 or 4 non-hematologic toxicity | First | 75 | 4.5 |
|  | Second | 50 | 3.0 |
|  | Third | Discontinue treatment* | |
| Grade 4 cutaneous toxicity, diarrhoea or mucositis | First | Discontinue treatment* | |

ANC, absolute neutrophil count; AUC, area under the concentration curve. *If an adverse event that requires dose reduction recurs after the dose has been reduced twice, treatment should generally be discontinued, unless, at the discretion of the investigator, there is evidence of continuing benefit to the patient that outweighs the risk of recurrent toxicity. Re-escalation is not permitted at any time.
