# Supplementary material for: nab-Paclitaxel-Based Therapy in Underserved Patient Populations: The ABOUND.70+ Study in Elderly Patients With Advanced NSCLC
Source: Front Oncol. 2018 Jul 24;8:262. doi: 10.3389/fonc.2018.00262 (PMC6066531; doi:10.3389/fonc.2018.00262)
Supplement: Supplementary file 3 [file Table_2.docx]

***Supplemental Table 2*:** Common (≥ 15% of patients) baseline comorbidities

| **System Organ Class/Preferred Term** | **21d Arm**  **(n = 71)** | **21d+break Arm**  **(n = 72)** |
| --- | --- | --- |
| Patients with ≥ 1 active comorbidity | 71 (100) | 72 (100) |
| Respiratory, Thoracic and Mediastinal Disorders | 57 (80.3) | 59 (81.9) |
| Chronic Obstructive Pulmonary Disease | 37 (52.1) | 26 (36.1) |
| Cough | 25 (35.2) | 26 (36.1) |
| Dyspnoea | 24 (33.8) | 22 (30.6) |
| Metabolism and Nutrition Disorders | 59 (83.1) | 53 (73.6) |
| Hyperlipidaemia | 27 (38.0) | 20 (27.8) |
| Decreased Appetite | 17 (23.9) | 17 (23.6) |
| Hypercholesterolaemia | 15 (21.1) | 9 (12.5) |
| Type 2 Diabetes Mellitus | 13 (18.3) | 11 (15.3) |
| Vascular Disorders | 54 (76.1) | 52 (72.2) |
| Hypertension | 48 (67.6) | 49 (68.1) |
| Musculoskeletal and Connective Tissue Disorders | 46 (64.8) | 46 (63.9) |
| Back Pain | 15 (21.1) | 16 (22.2) |
| Osteoarthritis | 14 (19.7) | 10 (13.9) |
| Gastrointestinal Disorders | 44 (62.0) | 41 (56.9) |
| Gastrooesophageal Reflux Disease | 20 (28.2) | 17 (23.6) |
| Constipation | 17 (23.9) | 19 (26.4) |
| General Disorders and Administration Site Conditions | 36 (50.7) | 39 (54.2) |
| Fatigue | 29 (40.8) | 30 (41.7) |
| Psychiatric Disorders | 41 (57.7) | 30 (41.7) |
| Anxiety | 26 (36.6) | 16 (22.2) |
| Insomnia | 22 (31.0) | 14 (19.4) |
| Depression | 17 (23.9) | 9 (12.5) |
| Cardiac Disorders | 27 (38.0) | 22 (30.6) |
| Coronary Artery Disease | 12 (16.9) | 11 (15.3) |
| Endocrine Disorders | 16 (22.5) | 18 (25.0) |
| Hypothyroidism | 15 (21.1) | 16 (22.2) |
| Blood and Lymphatic System Disorders | 13 (18.3) | 16 (22.2) |
| Anaemia | 11 (15.5) | 14 (19.4) |
