# Supplementary material for: nab-Paclitaxel-Based Therapy in Underserved Patient Populations: The ABOUND.70+ Study in Elderly Patients With Advanced NSCLC
Source: Front Oncol. 2018 Jul 24;8:262. doi: 10.3389/fonc.2018.00262 (PMC6066531; doi:10.3389/fonc.2018.00262)
Supplement: Supplementary file 4 [file Table_3.docx]

***Supplemental Table 3***: Common (≥ 15%) concomitant medications

| **World Health Organization Drug Class** | **21d Arm**  **(n = 71)** | **21d+break Arm**  **(n = 72)** |
| --- | --- | --- |
| Subjects with any concomitant medication | 71 (100) | 72 (100) |
| Antiemetics and antinauseants | 64 (90.1) | 65 (90.3) |
| Analgesics | 51 (71.8) | 53 (73.6) |
| Corticosteroids for systemic use | 49 (69.0) | 49 (68.1) |
| Antithrombotic agents | 42 (59.2) | 50 (69.4) |
| Lipid-modifying agents | 46 (64.8) | 37 (51.4) |
| Antibacterials for systemic use | 37 (52.1) | 40 (55.6) |
| Drugs for obstructive airway diseases | 38 (53.5) | 38 (52.8) |
| Mineral supplements | 36 (50.7) | 40 (55.6) |
| Drugs for acid-related disorders | 36 (50.7) | 38 (52.8) |
| Psycholeptics | 38 (53.5) | 35 (48.6) |
| Vitamins | 33 (46.5) | 37 (51.4) |
| Drugs for constipation | 35 (49.3) | 31 (43.1) |
| Blood substitutes and perfusion solutions | 29 (40.8) | 32 (44.4) |
| Agents acting on the renin-angiotensin system | 26 (36.6) | 31 (43.1) |
| Beta-blocking agents | 23 (32.4) | 32 (44.4) |
| Antianemic preparations | 24 (33.8) | 27 (37.5) |
| Diuretics | 24 (33.8) | 24 (33.3) |
| Antihistamines for systemic use | 22 (31.0) | 23 (31.9) |
| Antidiarrheals, intestinal antiinflammatory/antiinfective agents | 27 (38.0) | 17 (23.6) |
| Calcium channel blockers | 19 (26.8) | 23 (31.9) |
| Psychoanaleptics | 24 (33.8) | 16 (22.2) |
| Cough and cold preparations | 21 (29.6) | 17 (23.6) |
| Drugs used in diabetes | 18 (25.4) | 18 (25.0) |
| Immunostimulants | 15 (21.1) | 20 (27.8) |
| Antiinflammatory and antirheumatic products | 15 (21.1) | 18 (25.0) |
| Thyroid therapy | 15 (21.1) | 17 (23.6) |
| Stomatological preparations | 11 (15.5) | 13 (18.1) |
| Urologicals | 13 (18.3) | 10 (13.9) |
